# Challenges and obstacles implementing evidence-based food fortification policy in a high-income country

**DOI:** 10.3389/fpubh.2023.1052314

**Published:** 2023-03-16

**Authors:** Ronit Endevelt, Theodore H. Tulchinsky, Ziva Stahl, Zohar Mor, Nadav Davidovitch, Hagai Levine, Aron M. Troen

**Affiliations:** ^1^The Israel Ministry of Health, Jerusalem, Israel; ^2^School of Public Health, Faculty of Welfare and Health Sciences, Haifa University, Haifa, Israel; ^3^Department of Public Health, Ashkelon Academic College, Ashkelon, Israel; ^4^Department of Health Systems Management, Faculty of Health Sciences, Ben Gurion University of the Negev, Beer Sheva, Israel; ^5^The Hebrew University–Hadassah Medical Center Braun School of Public Health & Community Medicine, The Hebrew University of Jerusalem Faculty of Medicine, Jerusalem, Israel; ^6^The School of Nutritional Sciences and Institute of Biochemistry Food and Nutrition Science, The Robert H. Smith Faculty of Agriculture Food and Environment, The Hebrew University of Jerusalem, Jerusalem, Israel

**Keywords:** folate (folic acid), iodine, micronutrients, vitamin B12, calcium, vitamin D, NTD (neural tube defect), fortification

## Abstract

The World Health Organization (WHO) recognizes food fortification as one of the most cost-effective and beneficial public health measures available. Mass fortification policies and regulations can reduce health disparities, including in high-income countries, by improving micronutrient intake among food-insecure or high-risk populations without changing their diet or behavior. While international health organizations have traditionally prioritized technical assistance and grants to medium and low-income countries, it is important to recognize that micronutrient deficiencies may also pose an important yet underappreciated public health problem in many high-income countries. Nevertheless, some high-income countries, including Israel, have been slow to adopt fortification, due to a variety of scientific, technological, regulatory, and political barriers. Overcoming these barriers requires an exchange of knowledge and expertise among the all stakeholders to achieve cooperation and broad public acceptance within countries. Similarly, sharing the experience of countries where the matter is in play may help inform efforts to advance fortification globally. Here we share a perspective on progress and barriers to achieve this goal in Israel, to inform efforts made to avoid the regrettable waste of unrealized human potential from prevalent yet preventable nutrient deficiency conditions, in Israel and beyond.

## Perspective

Food fortification has been a safe and cost-effective method of preventing prevalent micronutrient deficiencies for over a century (1, 2). As of 2020, 143 countries around the world have adopted mandatory food fortification policies (3). Along with vaccinations, the World Health Organization (WHO) recognizes food fortification as one of the most cost-effective and beneficial public health measures available, and as a safe, effective, and inexpensive public health measure to prevent the harms associated with pernicious micronutrient deficiencies (4). Mass fortification can reduce health disparities including in high-income countries by improving micronutrient intake among food-insecure or high-risk populations without changing their diet or behavior (1, 5–8). Indeed, on January 31, 2023, the World Health Organization Executive Board decided to recommend that the World Health Assembly adopt a resolution calling to accelerate efforts for preventing micronutrient deficiencies and their consequences, including spina bifida and other neural tube defects, through safe and effective food fortification (9). The Executive Board recommendation is supported by Australia, Brazil, Canada, Chile, Colombia, Ecuador, Malaysia, Paraguay, the European Union and its 27 Member States, and by Israel. Nevertheless, some high-income countries, including in Europe and Israel, have been slow to adopt fortification.

Growing evidence indicates that micronutrient deficiencies in Israel present a significant public health challenge. Over the past two decades, the Israel Ministry of Health (MOH) established three panels, in 1996, 2010 and 2015; all recommended mandating food fortification. The latest panel recommended fortifying salt with iodine, milk with vitamin D and flour with iron, B-complex, including but not limited to folic acid and vitamin B12, using the Canadian approach of mandatory fortification, accompanied by national biomonitoring of the population's micronutrient status. This as recommended by the WHO and public health best practice (2, 4, 10–13). In late 2018, the Minister of Health, and MOH Director-General, endorsed the recommendations and authorized steps to implement appropriate regulations (14). A subsequent Regulatory Impact Assessment (RIA) recommended harmonizing Israeli requirements with the European rather than Canadian guidance on fortification, to require mandatory fortification of selected staples, while permitting industry-driven voluntary fortification of other food products, on condition that claims and marketing of voluntarily fortified food are restricted.

Although knowledge of the benefits of food fortification are over a century old, countries like Israel that wish to fortify their food face a variety of scientific, technological, regulatory, and political barriers, including achieving public acceptance (1, 15). For example, implementing a sustainable fortification program requires adequate evidence of the populations' nutritional status; knowledge of the population consumption patterns of the intended fortification vehicle (e.g. salt, milk, or flour); setting technical standards for fortified foods; a willing and technically capable food industry; consideration of the effect on trade export and import of fortified foods; enacting appropriate regulations and laws; establishing procedures for fortified food quality control and for monitoring the effect of the policy on the populations' nutrition and health; providing appropriate funding; and of course, a supportive public and public health community. All this requires an exchange of knowledge and expertise among the all stakeholders to achieve cooperation and broad public acceptance (1).

To this end, the MOH, Israel Association of Public Health Physicians and the Ashkelon Academic College, convened a conference in November 2019, to discuss evidence of micronutrient deficiencies in Israel and the MOH decision to fortify food [see (16)]. Participants included relevant stakeholders, government officials, the public health community, academic researchers, industry representatives, and the public at large. Presentations reviewed evidence of prevalent micronutrient deficiencies of vitamins A, C, D, and E, folate, iodine, calcium, magnesium and iron, and of neural tube defects, anemia, thyroid disease and rickets, based on dietary intake data (from Israel Center for Disease Control's MABAT (Nutrition and Health) surveys of representative samples of children, adults, and the elderly), clinical laboratory data (from the major Health Management Organizations), and academic studies (from the peer-reviewed medical literature). The meeting concluded with a round table exchange, indicating broad official and stakeholder support for fortifying food in Israel.

The main obstacles to mandate fortification are neither scientific nor technological (16). Rather, the challenge has been to gain the political motivation needed to draft and pass legislation designed to regulate, enforce and fund fortification, according to the specific health and nutrition needs of the Israeli population. The delay in doing so partly reflects concern over those significant regulatory, budgetary and political efforts that are necessary to give public health priority over competing interests. Unfortunately, the onset of the COVID-19 pandemic diverted MOH attention and resources from this important issue, and progress toward implementation has slowed. Nevertheless, at the urging of the MOH the Israel Standards Institute has begun to revise the salt and milk standards, to meet the intended requirements for local production and importation of iodized salt and iodine and vitamin D fortified milk (Personal communication, Endevelt, R.). Theoretically, market-driven, voluntary fortification might improve the dietary intakes of some Israelis, but extensive international experience shows that uncontrolled voluntary fortification is less effective, more prone to promote risk of excessive intake, and more likely to increase health disparities than mandatory, regulated food fortification. Thus, it is crucial that in addition to revising the food standards, Israel enacts regulations specifying the fortificants and food vehicles that must be fortified, while restricting the use of voluntary fortification for marketing purposes. The regulations should allow for periodic evaluation and adjustment to the fortification program to allow for possible changes in the populations' nutritional status and food intake. WHO guidelines on food fortification and extensive international experience can provide reassurance and guide Israel's response to these concerns.

Other concerns that mandatory fortification might restrict free trade, particularly with Europe, should be allayed by acknowledging that the World Trade Organization allows countries to create their own national food standards in accordance with the CODEX Alimentarius, and to legislate mandatory fortification of locally produced and imported food, when required for public health (17). Indeed, the European Union does not require harmonized food fortification standards. Rather, each European Member State regulates fortification based on the health needs of its own population: Ten states allow salt to be fortified with either potassium or sodium iodide (KI or NaI), two states permit potassium iodate only (KIO_3_), and nine states permit both iodide and iodate. In seven of twenty-five European states, salt iodization is mandatory (18). Furthermore, the required iodine concentrations differ between member states based on each population's iodine status, and none of this prevents European trade. Simply stated, trade considerations do not trump mandatory fortification, provided the legislation is necessary to ensure public health.

The MOH should also address the drafting of fortification legislation in the MOH work plan; perform cost-utility analyses; strengthen public support through information campaigns; make arrangements to sustain fortification; establish a steering committee with a mandate to design, oversee and enforce the program; and provide for the periodic monitoring of the population's nutrient intake and status.

The MOH should continue to place public health policy over competing interests and balance political pressures in order to affirm an effective and equitable policy. Doing so will ultimately improve the well-being of the Israeli public, by helping to lessen health and social disparities, reduce health system costs to the Israeli economy, and avoid the regrettable waste of unrealized human potential from these prevalent yet preventable deficiency conditions.

## International implications

While international health organizations have traditionally prioritized technical assistance and grants to medium and low-income countries, it is important to recognize that micronutrient deficiencies may also pose an important yet underappreciated public health problem in many high-income countries. Thus, national governments of high-income countries should prioritize preventing this “silent hunger” that causes birth defects and negatively impacts child development and the realization of human potential and health at all ages. Countries that fortify their food can provide positive examples of public health best practice to those, that have yet to do so (4, 19, 20).

Based on this international experience with micronutrient deficiencies and fortification, and the current situation in Israel, we draw the following conclusions:

### With regard to Israel

1. Micronutrient deficiencies can and do occur in Israel at levels which may harm vulnerable groups in the population that require public health action. Excess risk micronutrient deficiencies may be prevented by eradicating their antecedent deficiencies through evidence-based, mass food fortification.
2. Mandate fortification of salt with iodine, milk with vitamin D, flour with iron, vitamin B-complex including folic acid and vitamin B12, making it an integral element in Israel's health-promoting nutrition policy.

### Global public health recommendations

3. Micronutrient intake and status of the general and high-risk populations should be monitored on a regular basis.
4. Health funds and the MOH must promote awareness of the vital role of micronutrients for vulnerable populations: women, pregnancies, newborns, children, adolescents, adults and the elderly.
5. Medical, nursing, nutrition professional training programs should place nutrition public health among the highest priority messaging and competencies required for their professional training.
6. High, medium, and low-income countries should all be encouraged to consider mandatory fortification of common foods to promote health of their populations. The ongoing COVID-19 pandemic underscores the urgency of these measures.
7. WHO. UNICEF, UNDP, World Bank and other leading international organizations should make the elimination of the silent hunger of micronutrient deficiencies a high priority, and a key element of the Sustainable Development Goal of “Zero Hunger”.

## Data availability statement

The original contributions presented in the study are included in the article, further inquiries can be directed to the corresponding author.

## Author contributions

RE, TT, ZS, and AT conceived of and drafted this manuscript. All authors contributed to revising it critically for important intellectual content and approved of the final version to be published.

## References

[1] WHO and FAO. Allen L Benoist Bd Dary O Hurrell R editors. Guidelines on Food Fortification With Micronutrients. Geneva: World Health Organization and Food and Agricultural Organization of the United Nations (2006). Available online at: https://apps.who.int/iris/bitstream/10665/43412/1/9241594012_eng.pdf (accessed February 11, 2023).

[2] Neufeld LM Aaron GJ Garrett GS Baker SK Dary O Van Ameringen M. Food fortification for impact: a data-driven approach. Bull World Health Organ. (2016) 94:631–2. 10.2471/BLT.15.164756 27516642 PMC4969988

[3] *Global Fortification Data Exchange*. (2022). Available online at: https://fortificationdata.org/chart-year-when-food-fortification-mandated/; https://fortificationdata.org/full-gfdx-datasets/ (accessed February 11, 2023).

[4] WHO. Mobilizing Ambitious and Impactful Commitments for Mainstreaming Nutrition in Health Systems: Nutrition in universal health coverage, Global nutrition summit. Geneva: World Health Organization (2020).

[5] Baye K. Maximising benefits and minimising adverse effects of micronutrient interventions in low- and middle-income countries. Proc Nutr Soc. (2019) 78:540–6. 10.1017/S0029665119000557 30853033

[6] Csolle I Felso R Szabo E Metzendorf MI Schwingshackl L Ferenci T. Health outcomes associated with micronutrient-fortified complementary foods in infants and young children aged 6-23 months: a systematic review and meta-analysis. Lancet Child Adolesc Health. (2022) 6:533–44. 10.1016/S2352-4642(22)00147-X 35753314 PMC9279162

[7] Heidkamp RA Piwoz E Gillespie S Keats EC D'Alimonte MR Menon P. Mobilising evidence, data, and resources to achieve global maternal and child undernutrition targets and the Sustainable Development Goals: an agenda for action. Lancet. (2021) 397:1400–18. 10.1016/S0140-6736(21)00568-7 33691095

[8] Kancherla V Botto LD Rowe LA Shlobin NA Caceres A Arynchyna-Smith A. Preventing birth defects, saving lives, and promoting health equity: an urgent call to action for universal mandatory food fortification with folic acid. Lancet Glob Health. (2022) 10:e1053–7. 10.1016/S2214-109X(22)00213-3 35617975

[9] WHO Executive Board. Accelerating Efforts for Preventing Micronutrient Deficiencies Their Consequences, Including Spina Bifida Other Neural Tube Defects, Through Safe Effective Food Fortification. Draft decision proposed by Australia, Brazil, Canada, Chile, Colombia, Ecuador, European Union its 27 Member States, Israel, Malaysia Paraguay (2023). Available online at: https://apps.who.int/gb/ebwha/pdf_files/EB152/B152_CONF5-en.pdf (accessed February 11, 2023).

[10] Calvo MS Whiting SJ Barton CN. Vitamin D fortification in the United States and Canada: current status and data needs. Am J Clin Nutr. (2004) 80 (Suppl. 6):1710S−6S. 10.1093/ajcn/80.6.1710S 15585792

[11] Itkonen ST Erkkola M Lamberg-Allardt CJE. Vitamin D fortification of fluid milk products and their contribution to vitamin D intake and vitamin D status in observational studies-a review. Nutrients. (2018) 10:1054. 10.3390/nu10081054 30096919 PMC6116165

[12] Morris JK Addor MC Ballardini E Barisic I Barrachina-Bonet L Braz P. Prevention of neural tube defects in Europe: a public health failure. Front Pediatr. (2021) 9:647038. 10.3389/fped.2021.647038 34249803 PMC8264257

[13] Zimmermann MB Andersson M. Global endocrinology: global perspectives in endocrinology: coverage of iodized salt programs and iodine status in 2020. Eur J Endocrinol. (2021) 185:R13–21. 10.1530/EJE-21-0171 33989173 PMC8240726

[14] MOH Bureau of the Director General. Meeting summary of meeting on Food Fortification held on October 14, 2018. Internal Communication. Jerusalem: Ministry of Health.

[15] Dary O Mora JO. Food fortification: technological aspects. In:Caballero B Allen L Prentice A, editors. Encyclopedia of Human Nutrition. 3rd ed. Cambridge: Academic Press (2013). p. 306–14.

[16] Wolff-Sagy Y Rosen S Troen AM Tulchinsky TH. Conference Proceedings: Do Micronutrient Deficiency Conditions Exist in Israel in 2019? Challenges and Opportunities for Food Fortification 2019. Available online at: https://www.aac.ac.il/wp-content/uploads/ACC-MN-Conference-Proceedings-2019-Feb-19-12-FONT.pdf (accessed February 11, 2023).

[17] FAO WTO. Trade and Food Standards. Rome: The Food and Agriculture Organization of the United Nations and the World Trade Organization (2017).

[18] AKJ. Response of Arbeitskreis Jodmangel (AKJ), the German co-ordinating Committee on Preventing Iodine Deficiency Disorders (IDD) to EU Discussion Paper on the Setting of Maximum and Minimum Amounts for Vitamins and Minerals in Foodstuffs. European Commission Directorate General for Health and Food Safety (2016). Available online at: https://food.ec.europa.eu/system/files/2016-10/labelling_nutrition-supplements-responses-akj_en.pdf (accessed February 11, 2023).

[19] The krakow declaration on iodine: Tasks and responsibilities for prevention programs targeting iodine deficiency disorders. Eur Thyroid J. (2018) 7:201–4. 10.1159/000490143 30283738 PMC6140595

[20] FAO IFAD UNICEF WFP and WHO. The State of Food Security and Nutrition in the World 2021. Transforming Food Systems for Food Security, Improved Nutrition and Affordable Healthy Diets for All. Rome: FAO (2021).

